# Experimental study of diclofenac and its biliary metabolites on anastomotic healing

**DOI:** 10.1002/bjs5.63

**Published:** 2018-05-17

**Authors:** S. T. K. Yauw, R. M. L. M. Lomme, P. van den Broek, R. Greupink, F. G. M. Russel, H. van Goor

**Affiliations:** ^1^ Department of Surgery Radboud University Medical Centre Nijmegen The Netherlands; ^2^ Department of Pharmacology and Toxicology Radboud University Medical Centre Nijmegen The Netherlands

## Abstract

**Background:**

Diclofenac increases the risk of anastomotic leakage, but the underlying mechanism is unknown. As diclofenac is excreted largely as biliary metabolites, the aim of this study was to determine the effect of these metabolites on intestinal anastomoses.

**Methods:**

This was a randomized controlled blinded experiment using 210 male Wistar rats to assess the effect of ‘diclofenac bile’ on the anastomotic complication score, leak rate and anastomotic strength following oral and parenteral administration of diclofenac. Bile duct and duodenal catheterization techniques were used for diversion and replacement of bile, and biliary diclofenac metabolites were determined.

**Results:**

Replacement of control bile with diclofenac bile resulted in higher anastomotic complication scores (P = 0·006) and leakage in five of 18 animals, compared with one of 18 controls (P = 0·089). In turn, following oral diclofenac administration, replacement of diclofenac bile with control bile reduced anastomotic complications (P = 0·016). The leak rate was seven of 15 versus 13 of 17 without replacement (P = 0·127). After intramuscular administration of diclofenac, the reduction in anastomotic complications was not significant when bile was replaced with control bile (P = 0·283), but it was significant when bile was drained without replacement (P = 0·025). Diclofenac metabolites in bile peaked within 2 h after administration. Administration of diclofenac bile resulted in nearly undetectable plasma levels of diclofenac (mean(s.d.) 0·01(0·01) μg/ml) after 120 min. Following oral diclofenac, bile replacement with control bile did not affect the plasma concentration of diclofenac (0·12(0·08) μg/ml versus 0·10(0·05) μg/ml with diclofenac bile; P = 0·869).

**Conclusion:**

Altered bile composition as a result of diclofenac administration increases the ileal anastomotic complication rate in rats.

**Surgical relevance**

Diclofenac is thought to increase the risk of intestinal anastomotic leakage, but the underlying mechanism is unknown. Metabolites of diclofenac are excreted largely through bile.

Using bile transfer techniques, it is shown that exposure to bile from rats treated with diclofenac increases the number of anastomotic complications. That properties of bile and intraluminal drug concentrations are relevant for anastomotic healing is a novel perspective on the pathophysiology of leakage. Further clarification of related pathways could lead to targeted reduction of drug toxicity and reduction of the anastomotic leak rate.

## Introduction

Anastomotic leakage is the leading cause of morbidity and mortality after intestinal surgery, with leak rates varying from 3 to 14 per cent^1^. Non‐steroidal anti‐inflammatory drugs (NSAIDs) have been related to increased leak rates in both animal and retrospective human studies^2^, ^3^, ^4^. This is worrisome because NSAIDs are recommended as postoperative analgesics in current guidelines^5^. Clinical evidence is not considered sufficient to ban postoperative use of NSAIDs, as some studies do not report adverse effects^5^ ^6^. Further research is required to delineate the role of NSAIDs in clinical anastomotic leakage^4^ ^7^.

The main mechanism by which NSAIDs are believed to jeopardize anastomotic healing is the cyclo‐oxygenase (COX) 2 inhibitory action, which may cause suppression of inflammatory, proliferative or proangiogenic pathways relevant for normal wound healing^2^ ^8^. An alternative explanation could be direct toxicity of intraluminal factors resulting from NSAID treatment (such as drug metabolites and bile products). This is based on a similar relationship found between NSAIDs and small intestinal mucosal damage^9^, ^10^, ^11^.

It is hypothesized that the NSAID diclofenac may display its detrimental effect on bowel anastomoses through altered and toxic bile composition. At least three observations support this hypothesis. First, NSAID‐induced intestinal mucosal damage has been attributed to increased bile toxicity^12^, ^13^, ^14^, ^15^. Biliary excretion of toxic drug metabolites and reduced availability of protective phosphatidylcholine in bile due to competitive binding by NSAID metabolites appear to be responsible for the bile toxicity^9^ ^12^, ^13^ ^16^. Second, diclofenac causes leakage of anastomoses in the ileum and proximal colon, but not in the distal colon of the rat, which can be explained by the fact that most bile components are reabsorbed or converted to non‐toxic substances before reaching the distal colon^17^. Third, NSAIDs that are predominantly excreted through bile (such as diclofenac, celecoxib and carprofen) cause 80–100 per cent leakage of experimental ileal anastomoses, whereas naproxen, which is excreted almost entirely through urine in the rat and human, results in less than 15 per cent leakage^18^, ^19^, ^20^. In clinical practice, bile toxicity could pose a threat to a large number of patients, as it could be harmful for both small and large bowel anastomoses, considering the altered physiology following partial colectomy.

The aim of this study was to investigate the effect of altered bile composition induced by diclofenac on small bowel anastomotic healing in rats using bile drainage and bile replacement techniques.

## Methods

The study was carried out according to the ARRIVE guidelines^21^. It was approved by the Animal Ethics Committee (AEC number 2013‐171) and performed in the Central Animal Laboratory of Radboud University.

### Animals

In total, 210 adult male Wistar rats (mean(s.d.) weight 313(25) g; Harlan, Horst, The Netherlands) were used (138 to assess anastomotic healing and 72 to donate bile). The animals were acclimatized to laboratory conditions and initially housed, two per cage, at 22–23°C with a 12‐h day cycle, and free access to standard rodent chow (Ssniff^®^ R/M‐H; Bio Services, Uden, The Netherlands) and acidified tap water throughout the experiment. The cages were enriched with a rat retreat and nesting material, and were placed randomly on the shelves. All animals were checked at least twice daily. Humane endpoints were defined; animals were killed if they showed signs of severe discomfort (such as weight loss greater than 20 per cent, distended abdomen or severely reduced activity).

### Materials

Diclofenac sodium (DCF) (Cayman Chemical, Ann Arbor, Michigan, USA) dissolved in 0·1 per cent polysorbate in saline (sodium chloride 0·9 per cent) was used for administration by oral gavage, and as a standard for high‐performance liquid chromatography (HPLC) and liquid chromatography–mass spectrometry (LC–MS) analyses. DCF injection fluid (25 mg/ml; Centrafarm, Etten‐Leur, The Netherlands) diluted with saline to 3·33 mg/ml was used for intramuscular injections. 4‐Hydroxydiclofenac (4OH‐DCF) and 5‐hydroxydiclofenac (5OH‐DCF) (Toronto Research Chemicals, Toronto, Ontario, Canada), and diclofenac acyl‐β‐glucuronide (DAG) (LGC Standards, Wesel, Germany) were used as standards for LC–MS analyses, and diclofenac‐d_4_ (phenyl‐d_4_‐acetic) (J. H. Ritmeester, Nieuwegein, The Netherlands) was used as an internal standard for bile and plasma.

### Study design

**Figure 1:**
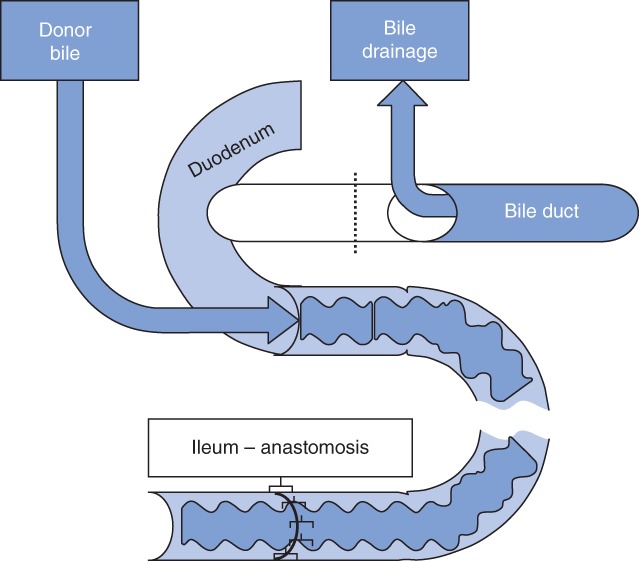
Schematic illustration of the experimental model used in experiments 1 and 3. In experiment 2, in groups IM+B0 and IM−B0, no bile was reinfused. IM, intramuscular; +, with diclofenac; −, without diclofenac; B0, bile drained and not returned

The study consisted of three experiments (*Table* 1). A distal ileum anastomosis model was used in all experiments and rats had surgery on day 0; anastomotic healing was assessed after killing on day 3^17^. This interval was used because most diclofenac‐induced leakage occurs before day 3, when the anastomosis is at its weakest, and postponing killing would increase suffering^20^. Rats received 1·5 mg/kg diclofenac twice daily either orally (O+) or intramuscularly (IM+), or they received no oral or intramuscular diclofenac (O− or IM−). Cannulation of the common bile duct (CBD) and the duodenum was performed to drain and infuse bile respectively (*Fig*. 1). Allocation to groups was done randomly by using the online tool at http://www.randomization.com.

**Table 1:** Experimental setup and groups

Group name	No. in group*	Diclofenac administration	Bile drainage and replacement
Experiment 1			
O−B−	18	None	Bile drained and replaced by ‘control bile’ from donor rats
O−B+	18	None	Bile drained and replaced by ‘diclofenac bile’ from donor rats
O+B−	15^1,2,3^	Oral	Bile drained and replaced by ‘control bile’ from donor rats
O+B+	17^3^	Oral	Bile drained and replaced by ‘diclofenac bile’ from donor rats
Experiment 2			
IM+B=	18	Intramuscular	No bile drainage or reinfusion
IM+B0	12	Intramuscular	Bile drained; no bile reinfused
IM−B0	11^4^	None	Bile drained; no bile reinfused
Experiment 3			
IM+B+	12	Intramuscular	Bile drained and reinfused instantly (rat receives its own bile)
IM+B−	10^4,5^	Intramuscular	Bile replaced with ‘control bile’ from donors

* Each superscript number denotes an animal that died during or just after operation. These were not included in the statistical analysis. Causes of death were:

^1^intraoperative bleeding of portal vein;

^2^anaesthetic overdose;

^3^catheter displacement;

^4^unknown cause;

^5^failure of bile duct cannulation.

In experiment 1, 72 rats were allocated to four groups of 18 each, and 72 rats were used as matched bile donors. In group O−B−, no diclofenac was administered and bile was drained and replaced by control bile from matched donor rats. In group O−B+ rats, the bile was replaced by ‘diclofenac bile’ from donor rats that had also received 1·5 mg/kg diclofenac orally twice daily for 3 days. Rats in group O+B− were administered diclofenac orally, and their bile was replaced with control bile^17^. Rats in group O+B+ were given diclofenac orally and received diclofenac bile from donors.

In experiments 2 and 3, a parenteral route of diclofenac administration was used to explore the effect of avoiding exposure of the gut to drug molecules as a direct result of oral administration^13^ ^14^. In experiment 2, 42 rats were allocated to three groups. Rats in group IM+B= (n = 18) received diclofenac intramuscularly, and no cannulations of the bile duct or duodenum were done. In groups IM+B0 and IM−B0 (n = 12 animals each), bile was drained completely without replacing it to prevent diclofenac metabolites and bile salts from entering the gut. In experiment 3, rats were given diclofenac intramuscularly and either received their own diclofenac bile (group IM+B+; n = 12) or bile was replaced with control bile from donor rats (group IM+B−; n = 12).

### Operative procedures

All operations were done by two experienced researchers. For analgesia, 0·02 mg/kg buprenorphine (Temgesic^®^; Schering‐Plough, Houten, The Netherlands) was given subcutaneously every 12 h, starting 1 h before the operation until 48 h afterwards. No antibiotics were given and animals were not fasted. Anaesthesia was with isoflurane (Abbott, Hoofddrop, The Netherlands), 5 per cent for induction and 2·5–3·5 per cent for maintenance, in a 1 : 1 mixture of oxygen and pressurized air. Rats were prepared by shaving, skin disinfection with iodine and sterile covers, and then operated on through a 4–5‐cm midline laparotomy using strict aseptic technique under a microscope (Wild M650; Wild Heerbrugg, Gais, Switzerland, at 10–16× magnification). Body temperature was kept at 38°C with a heating pad and lamp.

The procedures started with cannulation of the CBD to drain bile and placement of a catheter in the duodenum to readminister bile, if applicable. This was followed by construction of the bowel anastomosis. The laparotomy wound was closed with a running suture, 3/0 Vicryl^®^ (Ethicon, Amersfoort, The Netherlands) for the fascia and staples for the skin.

### Bile drainage and infusion techniques

The CBD was ligated with 7/0 silk (Pearsalls, Taunton, UK) just before it enters the tail of the pancreas, and a V‐shaped hole was made in the duct with iridectomy scissors proximal to the ligation. A custom‐made 3‐Fr polyurethane cannula (BTPU‐040; Instech Laboratories, Plymouth Meeting, Pennsylvania, USA), 20 cm in length, and a silicone ring (BTSIL‐047; Instech Laboratories) at 7 and 50 mm from the sharpened tip, was inserted into the bile duct and fixed with 7/0 silk proximal and distal to the silicon ring at 7 mm. A similar, but blunt, cannula was inserted through a purse‐string suture (6/0 Vicryl^®^; Ethicon, Norderstedt, Germany) in the duodenum. Cannulas were fixed to the abdominal wall (6/0 Vicryl^®^), tunnelled through the rectus muscle and subcutaneously towards the back between the scapulas, and then connected to a harness (VAHD95AB or VAHD115AB; Instech Laboratories). In turn, the harness was connected to a tether and swivel system (VAH95T+375/22PS or VAHD115T+375/D/22 with BTCOEX‐22 tubing; Instech Laboratories), through which the bile was transferred to 50‐ml tubes in a cooled (0–10°C) polystyrene box. When attached to the swivel, rats were still able to move within the entire cage and use nesting material.

In experiment 1, the tube with donor bile was changed twice daily and kept at 4°C until reinfusion the next day. Because the donor rats in experiment 1 also provided the control bile for experiment 3, and this experiment was conducted later, the bile was kept at −80°C until reinfusion. Because it involved control bile, there was no concern for loss of toxicity. Bile was infused into the duodenal cannula at room temperature (22–23°C) using a 20‐ml syringe and an infusion pump (IVAC Medical Systems, San Diego, California, USA) at a rate of 0·7–1·0 ml/h.

### Intestinal anastomosis

Anastomoses were created as described previously^17^. Briefly, 1 cm of ileum was resected at 15 cm proximal to the caecum. An inverted anastomosis was made with a single layer of eight interrupted (Lembert) sutures (8/0 Ethilon^®^; Ethicon, Norderstedt, Germany). After operation, rats were housed individually because they were attached to the swivel system.

### Assessment of outcome

Animals were killed by asphyxiation with a mixture of 95 per cent carbon dioxide and 5 per cent oxygen, and thereafter 100 per cent carbon dioxide (donor rats), or by cardiac or inferior vena cava puncture combined with cervical dislocation under general anaesthesia with isoflurane (experimental rats). A relaparotomy was done and the anastomosis was inspected macroscopically for anastomotic complications by a researcher blinded to the group randomization.

Signs were graded according to the anastomotic complication score: 0, no abnormality; 1, small (less than 2 mm) anastomotic abscess; 2, free pus or large abscess; 3, faecal peritonitis or visible dehiscence^17^. Grade 2 or 3 was considered an overt anastomotic leak.

Bursting pressure and breaking strength were assessed as described previously^17^. Briefly, bursting pressure (mmHg) was determined by infusing anastomotic segments with blue fluid and measuring the maximum pressure sustained before leakage occurred. Subsequently, segments were pulled apart with a tensiometer and the highest force measured before rupture was recorded as breaking strength (newtons).

### Analysis of diclofenac excretion profile in bile

To determine whether and when diclofenac products are found in bile, bile samples from four donor rats were obtained fractionally every hour after a single dose of diclofenac 1·5 mg/kg orally and analysed by HPLC; for methods used see Appendix S1 (supporting information).

### Quantitation of diclofenac metabolites in bile and plasma

Bile samples were obtained as described above and analysed by LC–MS/MS to determine which diclofenac metabolites were most abundant, and when. Plasma samples were collected in experiment 1 to assess whether bile replacement with either diclofenac bile or control bile influenced plasma concentrations through the enterohepatic circulation. Samples of 1 ml were drawn from the tail vein on postoperative day 2, either at 60, 90 or 120 min after diclofenac administration. Any change in plasma level was expected to occur after 60 min, based on biliary excretion time. For a description of methods used, see Appendix S1 (supporting information).

### Statistical analysis

Sample size was determined with expected leak rates of 70–80 per cent in groups receiving diclofenac bile and 10–20 per cent in groups receiving control bile or no bile^17^. Anticipating analysis of leak rates with Fisher's exact test, and anticipating a larger loss to follow‐up in the first experiment, 18 animals per group were used in experiment 1 and in group IM+B= of experiment 2, and 12 animals in other groups. Absolute leak rates, and not anastomotic complication scores, were used because this facilitates better sample size calculations. The Mann–Whitney U test was used to compare complication scores, and an independent t test to compare strength. Spread of data is presented as the standard deviation. P < 0·050 was considered statistically significant. Statistical analysis was performed with IBM SPSS^®^ Statistics version 22 (IBM, Armonk, New York, USA).

## Results

### Animal mortality and welfare

One rat in group O+B+ and two in IM+B+ were killed before day 3 because of severe illness due to peritonitis (humane endpoint). Other rats had a normal postoperative recovery or had signs of illness starting on day 3, just before killing. Six rats (3 in group O−B−, 2 in group O+B−, and 1 in group O+B+) had to be anaesthetized to relieve cannula obstruction by manipulating the subcutaneous part of the catheter (leakage occurred in the 2 rats in group O+B−). Two rats (1 in group O−B− and 1 donor rat) required a relaparotomy to relieve intra‐abdominal cannula kinking.

Postoperative weight loss in experiment 1 was comparable between groups (mean(s.d.) 9·8(2·8) per cent). In experiment 2, weight loss was significantly higher in groups where bile was not replaced (IM+B0: 13·7(4·4) per cent; IM−B0: 14·8(3·0) per cent) compared with loss in group IM+B=, where bile was replaced (10·0(2·6) per cent; P = 0·007 and P < 0·001 respectively). In experiment 3, weight loss was higher in group IM+B− than in group IM+B+ (14·6(2·7) versus 8·3(3·0) per cent respectively; P < 0·001).

### Anastomotic complication score and leak rate

**Figure 2:**
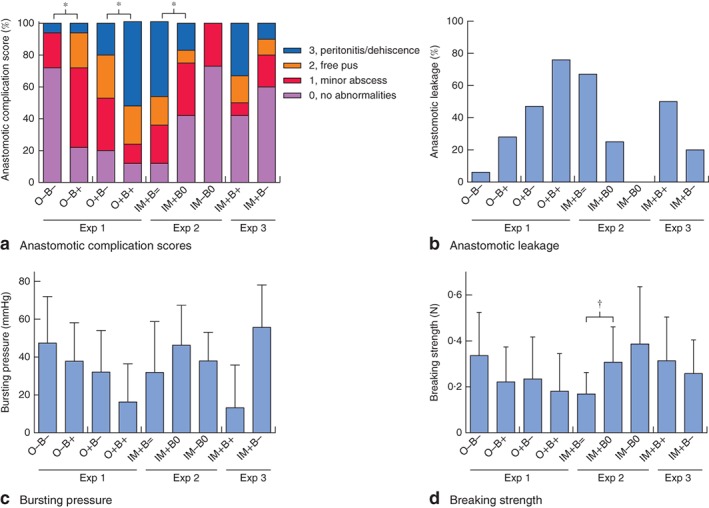
Anastomotic healing assessed by: **a** anastomotic complication scores, **b** anastomotic leakage, **c** bursting pressure (n = 5 in group IM+B+, n = 4 in group IM+B−, due to sensor failure) and **d** breaking strength. O, oral; IM, intramuscular; +, with diclofenac; −, without diclofenac; B+, bile from rats given diclofenac; B−, bile from control rats; B0, bile drained and not returned; B=, normal bile circulation. *P < 0·050 (Mann–Whitney U test); †P < 0·050 (independent t test)

In experiments 1, 2 and 3, anastomotic complication scores were the highest in ‘diclofenac bile’ groups (O−B+ versus O−B−, P = 0·006; O+B+ versus O+B−, P = 0·016; IM+B= versus IM+B0, P = 0·025; IM+B+ versus IM+B−, P = 0·283) (Fig. 2 a). Grades 2 and 3, which were considered leakage, occurred in five of 18 animals (28 per cent) after administration of diclofenac bile (O−B+), compared with one of 18 (6 per cent) with control bile (O−B−) (P = 0·089). Most leakage occurred in groups receiving oral or intramuscular diclofenac combined with diclofenac bile: 13 of 17 animals (76 per cent) in group O+B+ versus seven of 15 (47 per cent) in group O+B− (P = 0·127); 12 of 18 (67 per cent) in group IM+B= versus three of 12 (25 per cent) in group IM+B0 (P = 0·060); and six of 12 (50 per cent) in group IM+B+ versus two of ten (20 per cent) in group IM+B− (P = 0·117) (Fig. 2 b). No leakage occurred in group IM−B0 (P = 0·122 versus IM+B0; P = 0·001 versus IM+B=).

### Anastomotic strength

Bursting pressures corresponded with leak rates; values tended to be lower in groups with higher leak rates in experiments 1 and 3 (*Fig*. 2 *c*). In experiments 1 and 2, breaking strength was the highest in groups that received neither oral or intramuscular diclofenac nor diclofenac bile, with no significant differences (*Fig*. 2 *d*). A significant difference was observed between group IM+B= and group IM+B0 (mean(s.d.) 0·17(0·09) *versus* 0·31(0·15) N respectively; *P* = 0·006).

### Diclofenac excretion profile in bile

**Figure 3:**
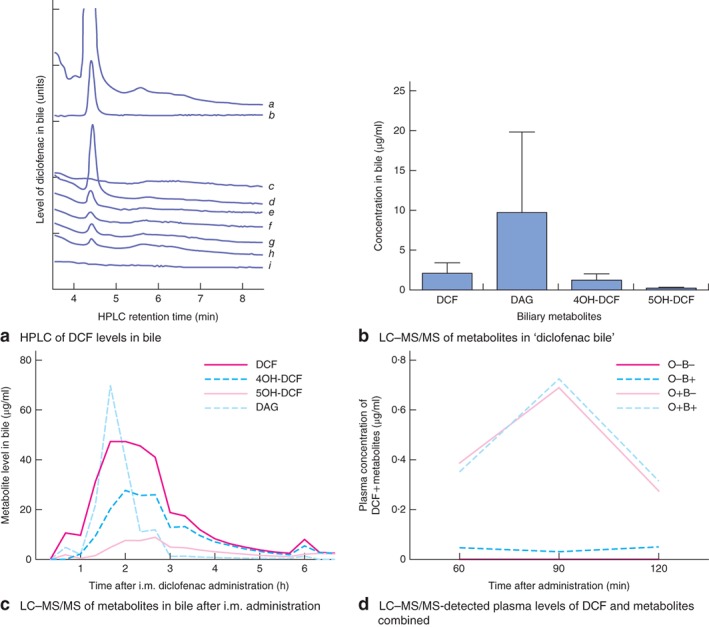
**a** High‐performance liquid chromatogram (HPLC) of hourly bile samples showing peak diclofenac levels within 1–2 h after oral administration: a, control bile with diclofenac sodium (DCF; 0·5 mg/ml) added; b, mobile phase with DCF (50 μg/ml); c, 0–1 h after administration; d, 1–2 h; e, 2–3 h; f, 3–4 h; g, 4–5 h; h, 5–6 h; i, control. **b** Liquid chromatography–mass spectrometry (LC–MS) or MS analysis of diclofenac metabolites in ‘diclofenac bile’ pooled over 2–8 h after oral administration (n = 9), showing diclofenac acyl‐β‐glucuronide (DAG) to be most abundant. **c** Quantitative LC–MS/MS analysis of metabolites in bile after intramuscular (i.m.) administration, showing DCF and DAG to be most abundant, and concentrations peaking at around 2 h (n = 2–6 per time point). **d** LC–MS/MS‐detected plasma levels of DCF, 4‐hydroxydiclofenac (4OH‐DCF) and 5‐hydroxydiclofenac (5OH‐DCF) combined, in samples from groups in experiment 1, showing that administration of diclofenac bile results in minimal plasma concentrations in group O−B+ and no discernible difference between O+B− and O+B+, with concentrations again dropping after 90 min; DAG could not be detected in plasma (n = 1–6). O, oral; +, with diclofenac; −, without diclofenac; B+, bile from rats given diclofenac; B−, bile from control rats

HPLC analysis revealed that diclofenac was detected in bile 1 h after oral administration, that levels peaked within 2 h, and that diclofenac remained detectable in bile at least 6 h after administration (*Fig*. 3 *a*).

### Bile production and analysis of diclofenac metabolites in bile

In the donor groups, bile production was comparable at a mean(s.d.) rate of 14·9(2·4) ml/day in control rats and 14·6(2·0) ml/day in diclofenac rats (*P* = 0·317). DAG was the most abundant metabolite in bile pooled over 2–8 h after oral administration (*Fig*. 3 *b*). Diclofenac metabolites were detected almost immediately after intramuscular administration, and levels of DAG, DCF and 4OH‐DCF peaked around 2 h after administration (*Fig*. 3 *c*).

### Analysis of diclofenac metabolites in plasma

Administration of ‘diclofenac bile’ in group O−B+ resulted in low mean(s.d.) plasma levels of DCF (0·01(0·01) μg/ml), 4OH‐DCF (0·01(0·01) μg/ml) and 5OH‐DCF (0·02(0·02) μg/ml) after 120 min. 5OH‐DCF was the most prominent metabolite found in plasma samples, with levels of 0·35(0·05) μg/ml in groups O+B− and O+B+ at 90 min, followed by DCF (0·26(0·02) μg/ml) and 4OH‐DCF (0·10(0·01) μg/ml). DAG was not detected.

The plasma DCF level in group O+B− at 120 min was comparable to that of group O+B+ (0·12(0·08) *versus* 0·10(0·05) μg/ml respectively; *P* = 0·869). The total concentration of DCF and metabolites combined is displayed in *Fig*. 3 *d*.

## Discussion

This study has demonstrated that increased bile toxicity due to diclofenac administration results in more anastomotic complications in rats. Replacing normal bile with ‘diclofenac bile’ disturbed anastomotic healing and, in turn, replacement of diclofenac bile with control bile reduced signs of anastomotic leakage following administration of diclofenac. It is suggested that the increase in bile toxicity is caused by biliary excretion of diclofenac metabolites and the subsequent increase of enteral drug and metabolite concentrations.

The composition of bile was shown to be altered rapidly by the presence of diclofenac metabolites after both oral and intramuscular administration. The metabolites, DAG and unconjugated DCF in particular, rapidly enter the gut via biliary excretion, regardless of the route of administration. It is hypothesized that these intraluminal molecules disturb anastomotic healing. In the present study, this was supported by the slightly higher leak rates in oral diclofenac groups compared with those in groups with intramuscular administration.

That altered bile composition and intraluminal drug molecules are detrimental in small bowel anastomosis corresponds with earlier findings^17^, where diclofenac caused leakage of anastomosis in the rat ileum and proximal colon, but not in the distal colon. Bile and drug concentrations are considerably lower in the distal colon and are known to cause most mucosal damage in the terminal ileum^13^. Furthermore, sustained release forms of diclofenac with absorption in more distal intestinal segments appear to divert the mucosal damage from the small to the large intestine^22^. Other studies^10^ ^12^, ^13^ of NSAID‐induced enteropathy have also attributed the damage to topical effects of drug molecules. Possible underlying mechanisms involve reuptake of active diclofenac molecules, initially causing mitochondrial injury and cell death, and, subsequently, initiation of an excessive inflammatory response by activation of Toll‐like receptor 4 on macrophages^11^ ^14^, ^23^. Another possibility is that the diclofenac molecules suppress secretion of mucus, making the anastomosis more vulnerable to leakage, as was recently suggested by Bosmans and colleagues^24^. Although diclofenac is excreted largely as DAG, this metabolite is reactivated by bacterial glucuronidase in the terminal ileum, where it can cause mucosal damage^12^ ^25^. In the present study, the acyl‐glucuronide metabolite (DAG) was also abundant in bile. Reactivation of this metabolite in the gut together with biliary excretion of unconjugated forms of diclofenac may lead to high enteral accumulation of potentially harmful drug molecules, which may jeopardize healing from the luminal side of the anastomosis^17^ ^25^.

The finding that healing was still disturbed when diclofenac was administered via the parenteral route and bile was either diverted or replaced by control bile may suggest a systemic role for diclofenac in causing anastomotic leakage. COX‐2 inhibition is considered the main feature of systemic diclofenac activity, and could affect essential inflammatory and proliferative pathways^8^ ^26^. Administration of ‘diclofenac bile’ had little impact on plasma levels of diclofenac through the enterohepatic circulation; thus the effect of diclofenac bile could not be explained by more potent systemic activity.

A valid model was used to study comprehensively the effect of diclofenac and bile composition on the most clinically relevant outcome: anastomotic complications. Comparison of leak severity showed significant differences; comparison of absolute leak rates did not. However, leak rate trends pointed in the same direction. Scoring leakage according to severity and clinical consequences is currently advised in the literature^27^. The lack of significance may have been the result of a slightly smaller effect size than expected from previous studies; the greatest difference was 42 per cent, compared with 50 per cent expected.

The daily amount of bile produced in rats of about 12–16 ml is comparable to the amount in humans of 250–1000 ml per day when adjusted for body surface area. Biliary excretion of diclofenac is poorly studied in humans and depends on genetic polymorphisms in drug metabolism, but is reported to be somewhat lower in humans than in rats^28^. After oral administration (the preferred prescription form of diclofenac) and excretion in bile, total diclofenac concentrations could reach comparable levels in the human gut for anastomotic injury.

Bile toxicity would have most effect on anastomoses that comprise the terminal ileum, where bile is reabsorbed and concentrations are higher, compared with large bowel anastomoses. However, considering the physiological changes that occur following partial or subtotal colectomy, bile toxicity could be comparably relevant for colorectal anastomoses. Clinical cohort studies^2^, ^3^, ^4^ ^8^ reporting an increased risk of leakage following NSAID administration involved both ileocolic and colorectal anastomoses.

The relevance of the properties of bile and possibly intraluminal drug concentrations in relation to anastomotic healing is a novel perspective on leakage pathophysiology, and underlying mechanisms should be further elucidated. Current evidence provided by retrospective clinical studies and animal models makes it ethically controversial to study NSAIDs in a prospective randomized trial^29^, and experimental studies are suggested to clarify further the role of intraluminal diclofenac metabolite concentrations, the influence of bile salts, and the role of other drug metabolites, such as those of ketorolac and ibuprofen, in leakage pathophysiology.

## Supporting information


**Appendix S1** Analysis of diclofenac excretion profile in bile by HPLC
