# Supplementary material for: Experimental study of diclofenac and its biliary metabolites on anastomotic healing
Source: BJS Open. 2018 May 17;2(4):220–8. doi: 10.1002/bjs5.63 (PMC6069360; doi:10.1002/bjs5.63)
Supplement: Supplementary file 1 — Appendix S1 Analysis of diclofenac excretion profile in bile by HPLC [file BJS5-2-220-s001.docx]

**BJS5_63**

**Experimental study of diclofenac and its biliary metabolites on anastomotic healing**

**S. T. K. Yauw, R. M. L. M. Lomme, P. van den Broek, R. Greupink, F. G. M. Russel and H. van Goor**

**Appendix S1** Analysis of diclofenac excretion profile in bile by HPLC

Bile samples of 4 donor rats were fractionally obtained hourly after a single dose of diclofenac (1.5 mg/kg orally) and initially stored at 4°C and within 4 hours stored at -80°C until use. Hourly samples were about 0.5 ml of bile. These samples were freeze dried and dissolved in 0.5 ml of mobile phase. Solutions were then centrifuged for 10 minutes at 3000 g and supernatant was collected and transferred to standard HPLC vials. Finally 10 µl was injected into the column.

The apparatus was a TSP HPLC model (Thermo scientific, Waltham, MA, USA), with a P4000 pump, AS3500 autosamples and UV2000 detector. Separation was performed using an Alltima^tm^ C18 5µ (15.0 mm x 4.6 mm) reversed phase column with a C18 (7.5 mm x 3 mm) guard column. The mobile phase consisted of a mixture of 55% methanol (Macron fine chemicals, Deventer, The Netherlands), 12% acetonitril (Boom, Meppel, The Netherlands) and 33% of a 1% acetic acid (Merck, Darmstadt, Germany) in water. The mobile phase was eluted at a flow of 1 ml/min and effluent was monitored at 282 nm.

*Quantitation of diclofenac metabolites in bile and plasma by LC-MS/MS*

Bile samples were obtained as described under HPLC analysis, and analyzed by LC-MS/MS to specify which diclofenac metabolites are most abundant and when. Plasma samples were collected in experiment 1 to assess if bile replacement with either D-bile or C-bile influenced plasma concentrations through the enterohepatic circulation. Samples of 1ml were drawn from the tail vein on postoperative day 2, either at 60, 90 or 120 minutes following diclofenac administration. Any change in plasma levels was expected to occur after 60 minutes based on biliary excretion time.

After dilution of the bile samples and removing proteins of the plasma samples, diclofenac and metabolite concentrations were determined with LC-MS/MS, using an Acquity UPLC (Waters, Milford, MA, USA) coupled to a Xevo TQ-S (Waters) triple quadrupole mass spectrometer. The compounds were separated on an HSS T3 analytical column (1.8 µm; 100 × 2.1 mm, Acquity UPLC®, Waters, Ireland). As internal standard we used deuterated diclofenac: Diclofenac-d4 (phenyl-d4-acetic) (JH Ritmeester, Nieuwegein, The Netherlands). The elution gradient was as follows: 0 min, 40% B; 7-10 min, 80% B; and 11-14 min, 40% B. Solvent A consisted of 0.1% formic acid in H_2_O and Solvent B consisted of 0.1% formic acid in Methanol. The column temperature was set at 40°C, and the flow rate was 300µl/min. The effluent from the UPLC was passed directly into the electrospray ion source. Positive electrospray ionization was achieved using nitrogen as a desolvation gas with ionization voltage at 1000 Volt. The source temperature was set at 550°C and argon was used as collision gas.

Detection of diclofenac (DCF), the internal standard and the metabolites was based on isolation of the protonated molecular ion, [M + H]+ and subsequent MS/MS fragmentations and a multi reaction monitoring (MRM) were carried out. The following MRM transitions were used: for DCF m/z 295.9 (parent ion) to m/z 215.0 and 250.0 (both product ions), for diclofenac-d_4_ m/z 300.0 (parent ion) to m/z 219.0 and 254.0, for both the hydroxyl-diclofenac (OH-DCF) m/z 311.9 to m/z 230.9 and 266.0 and for the diclofenac-acyl-glucuronide (DAG) m/z 471.8 to m/z 215.0 and 296.0.
